# Using the kidney failure risk equation to predict end-stage kidney disease in CKD patients of South Asian ethnicity: an external validation study

**DOI:** 10.1186/s41512-023-00157-x

**Published:** 2023-10-05

**Authors:** Francesca Maher, Lucy Teece, Rupert W. Major, Naomi Bradbury, James F. Medcalf, Nigel J. Brunskill, Sarah Booth, Laura J. Gray

**Affiliations:** 1 Department of Population Health Sciences, University of Leicester, Leicester, UK; 2 Department of Cardiovascular Sciences, University of Leicester, Leicester, UK; 3 John Walls Renal Unit, University Hospitals of Leicester NHS Trust, Leicester, UK

**Keywords:** Chronic kidney disease, Kidney failure risk equation, Ethnicity, External validation, Competing risks, Primary care cohort

## Abstract

**Background:**

The kidney failure risk equation (KFRE) predicts the 2- and 5-year risk of needing kidney replacement therapy (KRT) using four risk factors — age, sex, urine albumin-to-creatinine ratio (ACR) and creatinine-based estimated glomerular filtration rate (eGFR). Although the KFRE has been recalibrated in a UK cohort, this did not consider minority ethnic groups. Further validation of the KFRE in different ethnicities is a research priority. The KFRE also does not consider the competing risk of death, which may lead to overestimation of KRT risk. This study externally validates the KFRE for patients of South Asian ethnicity and compares methods for accounting for ethnicity and the competing event of death.

**Methods:**

Data were gathered from an established UK cohort containing 35,539 individuals diagnosed with chronic kidney disease. The KFRE was externally validated and updated in several ways taking into account ethnicity, using recognised methods for time-to-event data, including the competing risk of death. A clinical impact assessment compared the updated models through consideration of referrals made to secondary care.

**Results:**

The external validation showed the risk of KRT differed by ethnicity. Model validation performance improved when incorporating ethnicity and its interactions with ACR and eGFR as additional risk factors. Furthermore, accounting for the competing risk of death improved prediction. Using criteria of 5 years ≥ 5% predicted KRT risk, the competing risks model resulted in an extra 3 unnecessary referrals (0.59% increase) but identified an extra 1 KRT case (1.92% decrease) compared to the previous best model. Hybrid criteria of predicted risk using the competing risks model and *ACR* ≥ 70 mg/mmol should be used in referrals to secondary care.

**Conclusions:**

The accuracy of KFRE prediction improves when updated to consider South Asian ethnicity and to account for the competing risk of death. This may reduce unnecessary referrals whilst identifying risks of KRT and could further individualise the KFRE and improve its clinical utility. Further research should consider other ethnicities.

**Supplementary Information:**

The online version contains supplementary material available at 10.1186/s41512-023-00157-x.

## Background

Chronic kidney disease (CKD) has a global prevalence between 8 and 16% [1–4]. The number of patients reaching end-stage kidney disease (ESKD) and needing kidney replacement therapy (KRT) is estimated at 4.9–7.1 million worldwide [4]. In 2009–2010, it was estimated that CKD cost the NHS in England approximately £1.45 billion, with over half the cost due to ESKD [5]. As the prevalence of CKD increases, this cost will to continue to rise [4–6].

The desire to predict the risk of progression to KRT led to the development of the kidney failure risk equation (KFRE) [7]. The KFRE predicts the 2- and 5-year risk of needing KRT using an individual’s characteristics for patients with stages G3–G5 CKD (eGFR < 60 ml/min/1.73 ${\mathrm{m}}^{2}$). Three different models including four, six and eight risk factors were derived. The 4-variable model is used in clinical practice and contains age, sex, creatinine-based estimated glomerular filtration rate (eGFR) and urine albumin-to-creatinine ratio (ACR).

The KFRE was developed in North America, externally validated in a non-North American cohort [6] and was later recalibrated to a UK-specific cohort [8]. This UK-recalibrated KFRE is recommended for use in clinical practice by the National Institute for Health and Clinical Excellence (NICE) alongside other measures to determine whether referral for specialist assessment is required [9].

However, the UK recalibration did not assess the KFRE in patients of different ethnicities. There are known ethnic disparities in CKD progression; patients of South Asian ethnicity have a higher risk of needing KRT than white patients (subdistribution hazard ratio = 1.62) [3]. The UK South Asian population generally includes individuals with ancestry from Afghanistan, Bangladesh, Bhutan, India, the Maldives, Nepal, Pakistan or Sri Lanka [10]. This increased risk could be due to several factors. Diabetes is the most common cause of ESKD in developed countries, and the prevalence of diabetes is greater for South Asian individuals than white individuals [10–12]. Furthermore, South Asian individuals have a higher risk of hypertension, also associated with CKD. NICE has highlighted the lack of research on how the KFRE performs across ethnicities as a research priority [13].

An additional issue is that of competing risks. Competing risks are events which either prevent the occurrence or alter the probability of the outcome of interest [14–16]. For instance, CKD patients at risk of needing KRT are also at risk of death, with that risk increasing with age and frailty. Conventional approaches for developing risk equations do not consider competing risks, which can result in overestimation of the absolute risk of the outcome [14]. The KFRE does not account for the competing risk of death, although an external validation has shown that the 5-year KFRE overestimated the real-world risk of KRT [14, 17].

We aimed to extend the validation of the non-North American 5-year KFRE by the following:

1. Validating the equation for UK South Asian and white individuals
2. Producing and comparing updated risk equations which account for ethnicity, including evaluating a competing risks model
3. Assessing the effect on referral rates of implementing our updated KFRE in clinical practice by ethnicity

## Methods

This study is reported according to the TRIPOD guidelines (Supplementary Table 1) [18].

### Data

The data were established from the UK cohort study which performed an external validation of the non-North American KFRE (referred to as the original KFRE) irrespective of ethnicity [8]. Further information on data collection can be found in Major et al. [8]. Briefly, anonymised patient data were extracted from primary care practices participating in the study. All practices were based in 4 clinical commissioning groups (CCGs): East Leicestershire and Rutland, Leicester City, Nene (Northamptonshire) and West Leicestershire. The study time-period started on 1 December, 2004, and ended on 1 November 2016. The outcome of kidney failure was defined as ESKD needing to be treated by KRT within 5 years. KRT is defined as treatments including haemodialysis, haemofiltration, haemodiafiltration, peritoneal dialysis and kidney transplantation [9]. Patients were included in the cohort if a quantifiable urine proteinuria (ACR or protein-to-creatinine ratio) measurement had been recorded and if they had two eGFR values < 60 ml/min/1.73 ${\mathrm{m}}^{2}$, taken at least 90 days apart. The date of the proteinuria measurement became the date for estimating baseline risk using the KFRE and for beginning the follow-up period. eGFR was measured using the 2009 CKD-EPI equation [19]. All patients were followed up until the event outcome, death, end of the study period or early exit from the study due to another reason (e.g. leaving the practice).

Several predictors were recorded at baseline: age, sex, ACR, eGFR, diabetes mellitus (DM), heart failure, cardiovascular disease and hypertension. Ethnicity was also collected, though it was not used for analysis in the previous external validation. The majority of patients were of either white or South Asian ethnicity. The numbers of patients of other ethnicities were too small to analyse and were excluded, as were those with missing ethnicity.

### Sample size

Given the size of the South Asian cohort (2728), the calibration slope could be reported with a 95% confidence interval (CI) width of 0.475 [20]. For the white cohort (27,017), the calibration slope could be reported with a 95% CI width of 0.237. These were deemed acceptable.

The minimum sample size for developing a prognostic model which satisfies the criteria in Riley et al. was 4241 [21]. The models developed in this analysis used a sample size of 29,745, well above this requirement.

### Analysis

Analysis was performed using R (version 4.1.3). The dataset supporting the conclusions of this article is available in Figshare [DOI: 10.25392 https://doi.org/10.25392/leicester.data.9860807.v1] [8]. A complete case approach was taken given there were no missing data for any of the variables included in the analysis.

Differences between the two ethnicities were investigated by summarising baseline characteristics. The predicted 5-year risk of KRT was calculated at baseline for each individual using the original KFRE. Patients were then categorised into risk groups split at < 3%, 3–< 5%, 5–< 15%, 15–< 25%, 25–< 50% and ≥ 50%, as in previous validations [6, 8].

### External validation

Validation of the original KFRE (Supplementary Text 1) was performed by ethnicity. Discrimination was assessed using Harrell’s C-statistic with a bootstrapped 95% CI (using the percentile method). The model performance at 5 years was of primary interest, so observations were truncated at 5 years.

Calibration was summarised by calibration plots. Patients of each ethnicity were grouped using deciles of predicted risk, and average predicted versus observed risks for each risk group were plotted by ethnicity. Also, KRT event indicators were replaced by pseudo-observations and a smooth calibration curve plotted [22]. Calibration was then summarised quantitatively by the following performance measures:

- Calibration intercept assessed overall calibration; the calibration slope assessed the level of variation in the predictions [23]. The observed/expected (O/E) ratio gave calibration-in-the-large and an overall measure of model calibration.
- Brier score and the scaled Brier score assessed model fit.

### Model updating

Where calibration was poor, the model was updated using a variety of methods [18]. Poor calibration was determined by inspection of the plots and by calibration measures that differed from 1 (calibration slope, O/E ratio) and 0 (calibration intercept). The Shiny package was used to create an online R Shiny app to illustrate the calibration of the updated models for each ethnicity. Using the performance measures listed above, we compared the models described in Table 1.

**Table 1:** Model updating methods used in the analysis

*Model*	*Explanation*	*Reason for updating*
1	No change — original model	--
2a — White cohort 2b — South Asian cohort	Adjustment of the baseline risk/hazard	Miscalibration in the large; overall O/E ratio is not close to 1
3a — White cohort 3b — South Asian cohort	Method 1 + adjusting the magnitude of the linear predictor	Calibration slope not close to 1 — the regression coefficients are over/under-fitted
4	Method 2 + addition of ethnicity as a predictor	A new factor is found to be important to the model
5	Re-estimation of all regression coefficients + addition of ethnicity as a predictor	The weighting of the coefficients in the original model needed to be adjusted after inclusion of a new predictor
6	Method 4 + accounting for competing risks (Fine and Gray model)	Other events (which prevent the primary event from occurring)

Models 2a, 2b, 3a and 3b were created by updating the KFRE separately, using the corresponding ethnicity cohort. When developing a new model (i.e. models 5 and 6), the variables were scaled and centred, and ACR was log-transformed as according to the original KFRE to allow for comparison between the old and updated model coefficients

Model 5 was the result of developing a new Cox model. Interaction effects between ethnicity and the other predictors in the KFRE were considered. Each interaction was added individually to the model, and a likelihood ratio test was used to compare models. Interactions were included if the likelihood ratio test was statistically significant at the 95% level. No other predictors were considered for inclusion. The model was internally validated by bootstrapping and the optimism reported. The optimism-adjusted calibration slope was applied as a uniform shrinkage factor to reduce model overfitting.

### Competing risks

Prognostic models are typically validated utilising the same modelling assumptions used when they were developed. The KFRE does not consider the competing risk of death, and therefore, models 2–5 were first validated using the Kaplan-Meier as a measure of the observed risk. However, Rampsek et al. recommend accounting for competing risks during external validation if they are known to occur in a clinical setting [14].

We validated model 5 again using the Aalen-Johansen estimator to estimate the observed risk. Individuals were categorised into the pre-defined risk groups using risk predicted from model 5. Aalen-Johansen cumulative incidence curves were plotted by risk group for each ethnicity, accounting for the risk of a competing event, unlike the Kaplan-Meier [24]. They were compared to the complement of the Kaplan-Meier curves, to compare observed risk with and without accounting for competing risks.

A competing risk (Fine and Gray) model, model 6, was fitted with the same predictors as model 5 and was internally validated. The performance of models 5 and 6 were compared. The performance measures were adjusted for the presence of competing risks.

### Clinical impact

We evaluated the impact of models 2–6 against the NICE guidelines for referral to secondary care. Previously, the guidelines stated that an individual with CKD-EPI eGFR < 30 ml/min/1.73 ${\mathrm{m}}^{2}$ and/or *ACR* ≥ 70 mg/mmol should be referred to secondary care renal services [8]. The guidelines were updated in 2019 to include predicted risk ≥ 5% from the KFRE with *ACR* ≥ 70 mg/mmol as part of the referral criteria, due to the study by Major et al. [8]. As this study uses the same dataset, the referral criteria before 2019 were evaluated to see whether the same conclusions about updating the guidelines could be drawn when considering ethnicity.

The number of correct, incorrect and missed referrals in the UK dataset was found under the previous and the 2019 criteria for each model. The net benefit of each model was found for threshold probabilities between 1 and 12% and decision curves plotted. The assessment was conducted in the eligibility assessment cohort, i.e. only patients not previously known to secondary care.

## Results

### Data

The data included 35,539 individuals. A total of 5794 individuals were removed (5115 with missing ethnicity and 679 individuals with ethnicity other than white or South Asian). Overall, 29,745 participants were included in the analysis.

Patients of South Asian ethnicity were on average 6 years younger, had a greater ACR and had a higher proportion of DM than the white group (Table 2). In the white cohort, 290 KRT events and 5421 deaths occurred within 5 years. In the South Asian cohort, 104 KRT events and 220 deaths occurred within 5 years. There were 18,554 patients censored before 5 years. Of these, 6692 (36.1%) were deaths.

**Table 2:** Baseline characteristics, follow-up and outcomes of the cohort by ethnic group

	***White (*N* = 27017)***	***South Asian (*N* = 2728)***
***Age (years)***	76.6 ± 10.3	70.2 ± 11.6
***eGFR (ml/min/1.73 *** ${m}^{2}$***)***	48.0 ± 9.88	48.1 ± 10.6
***ACR (mg/mmol)***	3.10 (1.30, 7.70)	4.90 (1.60, 13.8)
***Sex***		
*Male*	11,300 (42)	1286 (47)
*Female*	15,717 (58)	1442 (53)
***Diabetes mellitus***	8251 (31)	1450 (53)
***Heart failure***	2558 (9)	209 (8)
***Cardiovascular disease***	8982 (33)	792 (29)
***Hypertension***	19,001 (70)	1902 (70)
***Follow-up and outcomes***		
***Follow-up time (years)***		
*Mean ± SD*	4.74 ± 2.52	5.66 ± 2.78
*Median (IQR)*	4.72 (2.76, 6.55)	5.79 (3.41, 8.18)
***Time to needing KRT (years)***		
*Mean ± SD*	3.46 ± 2.34	3.63 ± 2.27
*Median (IQR)*	3.16 (1.60, 4.94)	3.35 (1.83, 5.17)
***Deaths*** *Within 5-year follow-up*	5421 (20)	220 (8)
***Death rate*** *Per 1000 person-years*	59.6	31.0
***KRT events*** *Within 5-year follow-up*	290 (1)	104 (4)
***KRT rate*** *Per 1000 person-years*	2.95	9.20

Results are given as mean ± standard deviation, median (interquartile range) or number (%). Comparisons of the data to the development data are found in Major et al. [8]. *eGFR* Estimated glomerular filtration rate, *ACR* Albumin-to-creatinine ratio, *SD* Standard deviation, *IQR* Interquartile range, *KRT* Kidney replacement therapy

### External validation

Supplementary Fig. 1 shows the Kaplan-Meier curves for each ethnicity. The model discrimination at 5-year follow-up showed clear separation of risk between the risk groups.

**Fig. 1:**
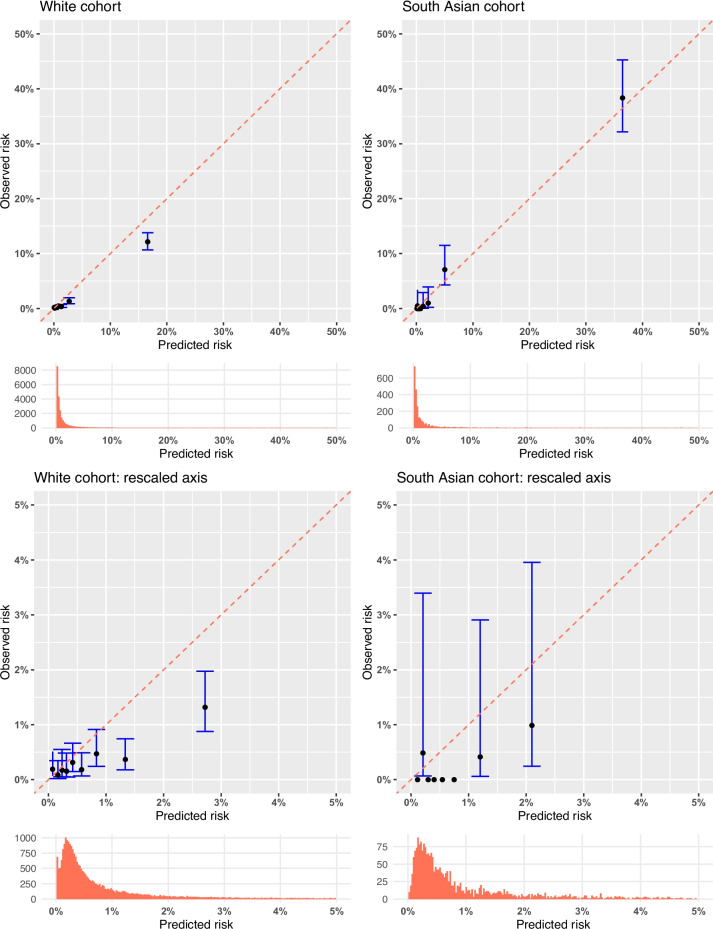
Calibration plots for predicted versus observed 5-year risk of KRT by ethnic group, alongside the distribution of the predictions. Individuals were categorised into risk groups using deciles of predicted risk according to the original non-North American KFRE. Underneath the plots are histograms of the distribution of the predicted risk. The dashed line indicates perfect calibration. The black dots represent the predicted and observed risk for each decile of risk group, with the blue bars showing the 95% confidence interval. The predicted risk of KRT in the cohort is low, so the majority of the risk groups are in the bottom left of the plots. The plots were thus truncated at 50% risk on the first row, and at 5% on the second row, to show the calibration more closely

**Fig. 2:**
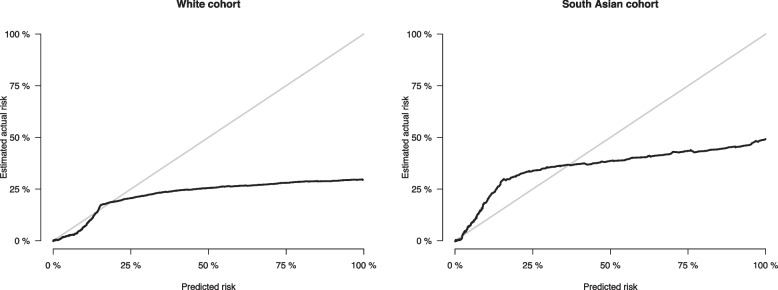
Calibration plots of predicted vs observed risk using pseudo-observations. Observations of risk that were very high were due to extreme ACR values, which skewed the pseudo-observations. A total of 96.9% of risks were < 15% in the white cohort, and 93.0% were < 15% in the South Asian cohort, so inspecting calibration at the higher risks is unreliable in this dataset

The model calibration for both ethnicities was evaluated using calibration plots, both with the original data (Fig. 1) and using pseudo-observations (Fig. 2). The model consistently over-predicted risk of KRT for those of white ethnicity, particularly at higher risks. Conversely, in the South Asian cohort, the model was over-fitted, and there was inconsistency in the miscalibration.

The external validation results are reported in Table 3. Harrell’s C-statistic was high for both ethnicities, similar to that reported for the original KFRE (0.88 (95% *CI*: 0.85, 0.91) [6]), showing excellent discrimination at a similar value to the pooled C-statistic in the development cohort. In the white cohort, the calibration slope was close to 1 (1.05, 95% *CI*: 0.920, 1.18), but the intercept was not close to 0 (−0.394, 95% *CI*: −0.555, −0.232), indicating the miscalibration was mainly in the large. In the South Asian cohort, the overall measure of calibration was good. However, the calibration slope indicated predicted risks were too extreme (0.843 (95% *CI*: 0.630, 0.995)).

**Table 3:** Values of the performance measures for each aspect of validation of the original KFRE

***Performance measure***	**White cohort (95%** *CI***)**	**South Asian cohort (95%** *CI***)**
***Discrimination***		
*C-index (up to 5 years)*	0.908 (0.887, 0.930)	0.954 (0.937, 0.971)
***Calibration***		
*O/E ratio*	0.623 (0.505, 0.740)	0.979 (0.789, 1.169)
*Calibration intercept*	–0.394 (–0.555, –0.232)	0.107 (–0.159, 0.373)
*Calibration slope*	1.05 (0.920, 1.18)	0.843 (0.690, 0.995)
***Overall fit***		
*Brier score*	0.011 (0.010, 0.012)	0.029 (0.022, 0.035)
*Scaled brier score (%)*	24.6 (16.6, 31.1)	34.7 (20.7, 45.4)

The 95% confidence intervals for the c-index and scaled brier score were calculated via bootstrapping with 1000 samples. *CI* Confidence interval, *O/E* ratio observed/expected ratio

### Model updating (models 2–4)

As calibration differed by ethnicity, the KFRE was updated separately by ethnicity (Supplementary Table 2, Supplementary Text 2). The updating methods gave a satisfactory increase in performance, but did not allow for re-estimation of the existing predictors, so a new model was developed.

### Developing a new model

Ethnicity interactions with ACR and eGFR improved model fit and were included in model 5 (Supplementary Table 3). The optimism was small for all performance measures (Table 5). The linear predictor was multiplied by the shrinkage factor, 1.015, and the baseline risk re-estimated. Model 5 showed an increased performance compared to models 2–4 across the performance measures and was well-fitted to the data (Table 4). A comparison of the calibration of each model by ethnicity and overall can be found at https://crsu.shinyapps.io/KFRE/.

**Table 4:** A comparison of model performance across models 2–5

***Performance measure***	Model 2a	Model 2b	Model 3a	Model 3b	Model 4		Model 5	
	White (95% CI)	South Asian (95% CI)	White (95% CI)	South Asian (95% CI)	White (95% CI)	South Asian (95% CI)	White (95% CI)	South Asian (95% CI)
***Discrimination***								
*C-index (up to 5 years)*	0.908 (0.886, 0.928)	0.954 (0.933, 0.968)	0.908 (0.886, 0.928)	0.954 (0.933, 0.968)	0.908 (0.886, 0.928)	0.954 (0.933, 0.968)	0.919 (0.898, 0.939)	0.957 (0.936, 0.974)
***Calibration***								
*O/E ratio*	0.938 (0.821, 1.06)	1.00 (0.815, 1.19)	0.946 (0.828, 1.06)	0.967 (0.778, 1.16)	0.938 (0.821, 1.06)	1.01 (0.820, 1.20)	1.12 (1.00, 1.24)	0.972 (0.782, 1.16)
*Calibration intercept*	0.094 (−0.067, 0.255)	0.146 (−0.120, 0.413)	0.062 (−0.101, 0.226)	0.151 (−0.103, 0.404)	0.096 (−0.065, 0.257)	0.153 (−0.113, 0.419)	0.227 (0.046, 0.409)	0.156 (−0.107, 0.419)
*Calibration slope*	1.05 (0.920, 1.18)	0.843 (0.690, 0.995)	1.02 (0.894, 1.15)	0.917 (0.751, 1.08)	1.05 (0.921, 1.18)	0.843 (0.691, 0.996)	0.905 (0.798, 1.01)	0.950 (0.752, 1.15)
***Overall fit***								
*Brier score*	0.011 (0.009, 0.012)	0.029 (0.023, 0.035)	0.011 (0.009, 0.012)	0.029 (0.023, 0.034)	0.011 (0.009, 0.012)	0.029 (0.023, 0.035)	0.010 (0.009, 0.012)	0.025 (0.019, 0.030)
*Scaled B*rier score* (%)*	25.4 (20.2, 29.3)	34.6 (20.7, 43.7)	25.6 (21.3, 29.4)	35.0 (23.6, 45.3)	25.4 (19.7, 29.8)	34.6 (21.8, 44.1)	27.4 (22.7, 31.6)	43.8 (31.9, 52.8)

Models 2a and 2b adjusted the baseline risk by ethnic group, models 3a and 3b rescaled the linear predictor and updated baseline risk for each group, model 4 included ethnicity as a predictor, and model 5 included ethnicity as a predictor and re-estimated the model coefficients. 95% confidence intervals were found via bootstrapping with 1000 simulations for the c-index and the scaled Brier score. *CI* confidence interval, *O/E* ratio observed/expected ratio

### Competing risks

The percentage of death was 26.6%, higher than the percentage of kidney failure events (1.60%). Supplementary Fig. 2 shows the 5-year risk of KRT for each risk group and overall, with and without competing risks.

A Fine and Gray model (model 6) which considered the competing risk of death was developed and internally validated (Table 5). The optimism was small for all performance measures.

**Table 5:** Internal validation of models 5 and 6

*Performance measure*	*Apparent performance*	*Optimism*	*Optimism-adjusted performance*
***Model 5***			
*C-index*	0.9313	0.0003	0.9310
*Overall O/E ratio*	1.025	0.002	1.023
*Calibration intercept*	0.276	0.00009	0.276
*Calibration slope*	1.025	0.010	1.015
*Brier score*	0.012	0.00004	0.012
*Scaled Brier* score* (%)*	31.3	0.27	31.0
***Model 6***			
*C-index*	0.9339	0.0003	0.9336
*Overall O/E ratio*	1.04	0.003	1.04
Calibration intercept	0.249	0.002	0.247
*Calibration slope*	0.956	0.007	0.948
*Brier score*	0.011	0.00007	0.011
*Scaled Brier s*core* (%)*	29.0	0.40	28.6

Internal validation completed via bootstrapping with 500 samples. The optimism-adjusted calibration slope can be applied as a uniform shrinkage factor to adjust the model coefficients. *O/E* observed/expected

Table 6 compares models 5 and 6 in a competing risks setting. Discrimination remained excellent across models. Model 5 performed significantly worse when accounting for competing risks, with the calibration slope significantly differing from 1. Model 6 had an improved performance in the competing risks setting — calibration measures were very close to 1, and the overall fit improved. Comparisons between model coefficients, calibration plots and predictions are given (Supplementary Table 4 and Supplementary Figs. 3 and 4).

**Table 6:** Model performance measures for models 5 and 6 in a competing risks setting

***Performance measure***	**Model 5 (95%** *CI***)**	**Model 6 (95%** *CI***)**
*Average predicted risk (%)*	1.61	1.55
*Average observed risk (%)*	1.56 (1.41, 1.72)	1.56 (1.41, 1.72)
***Discrimination***		
*C-index (up to 5 years)*	0.929 (0.912, 0.945)	0.932 (0.915, 0.946)
***Calibration***		
*O/E ratio*	0.973 (0.874, 1.07)	1.01 (0.913, 1.11)
Calibration intercept	0.035 (−0.111, 0.180)	0.286 (0.153, 0.420)
*Calibration slope*	0.886 (0.804, 0.969)	1.01 (0.916, 1.10)
***Overall fit***		
*Brier score*	0.011 (0.010, 0.012)	0.011 (0.010, 0.012)
*Scaled Brier* score* (%)*	29.4 (25.1, 34.5)	28.9 (25.4, 33.0)

Performance measures were adjusted for optimism by finding performance measures for the internally validated models. Also included are the overall mean observed and predicted risks. *CI* confidence interval, *O/E* observed/expected

The equation of the best model, model 6, is given in Supplementary Text 3.

### Clinical impact

**Fig. 3:**
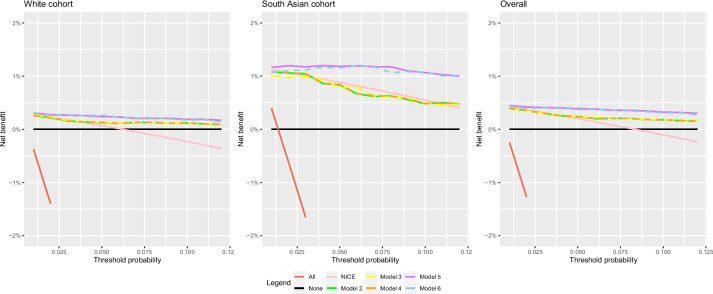
Net benefit of models 2–6, as well as the net benefit of the previous NICE guidelines (*eGFR* < 30 ml/min/1.73 ${\mathrm{m}}^{2}$, *ACR* ≥ 70 mg/mmol for referral)

In the South Asian cohort, models 2–4 had a lower net benefit than the NICE guidelines (Fig. 3). Using a risk threshold of 5%, models 2–4 outperformed the NICE guidelines in the white cohort.

Model 6 resulted in 227 less unnecessary referrals (a decrease of 30.8%) compared to the previous guidelines (Table 7). Missed KRT cases also decreased by 21.6%, from 37 to 29. In comparison to model 5, model 6 increased unnecessary referrals from 506 to 509 (0.59%) but increased correct referrals from 52 to 53 (1.92%). Increasing referrals by 3 identified an additional case of KRT.

**Table 7:** Number of total, correct, and unnecessary referrals and KRT cases missed when using ≥ 5% 5-year predicted KRT risk or *ACR* ≥ 70 mg/mmol as referral criteria for models 2–6, compared to previous NICE guidelines (referral if *eGFR* < 30 ml/min/1.73 ${m}^{2}$ and/or *ACR* ≥ 70 mg/mmol)

	*Correct referrals*	*Unnecessary referrals*	*Missed KRT cases*	*Total referrals*
***White (N = 11,596)***				
*NICE guidelines*	26 (4.09)	609 (96.0)	32 (55.2)	635
*Model 2*	29 (5.38)	510 (94.6)	29 (50.0)	539
*Model 3*	29 (5.50)	498 (94.5)	29 (50.0)	527
*Model 4*	29 (5.39)	509 (94.6)	29 (50.0)	538
*Model 5*	29 (7.63)	351 (92.4)	29 (50.0)	380
*Model 6*	30 (8.09)	341 (91.9)	28 (48.3)	371
***South Asian (N = 2124)***				
*NICE guidelines*	19 (13.0)	127 (87.0)	5 (20.8)	146
*Model 2*	23 (10.9)	188 (89.1)	1 (4.17)	211
*Model 3*	23 (10.2)	202 (89.8)	1 (4.17)	225
*Model 4*	23 (11.0)	186 (89.0)	1 (4.17)	209
*Model 5*	23 (12.9)	155 (87.1)	1 (4.17)	178
*Model 6*	23 (12.0)	168 (88.0)	1 (4.17)	191
***Overall (N = 13,720)***				
*NICE guidelines*	45 (5.76)	736 (94.0)	37 (45.1)	781
*Model 2*	52 (6.93)	698 (93.1)	30 (36.6)	750
*Model 3*	52 (6.91)	700 (93.1)	30 (36.6)	752
*Model 4*	52 (6.96)	695 (93.0)	30 (36.6)	747
*Model 5*	52 (9.32)	506 (90.7)	30 (36.6)	558
*Model 6*	53 (9.43)	509 (90.6)	29 (35.4)	562

Values given as number (percentage of total referrals) for correct and unnecessary referrals. Values given as number (percentage of total KRT cases within 5 years) for the missed KRT cases. Models 2a, 2b and 3a, 3b have been combined when giving an assessment of the whole cohort. The eligibility assessment cohort was used, i.e. those who are not previously known to secondary care. *NICE* National Institute for Clinical Excellence, *KRT* kidney replacement therapy

## Discussion

We found that miscalibration was present in the non-North American KFRE, and it differed by ethnicity. There was general over-prediction of risk in the white cohort, whereas in the South Asian cohort, the opposite was true, and under-prediction of risk occurred. Assessing the new model in a competing risks setting resulted in a change in risk of needing KRT, so the KFRE also needs to account for competing risks.

The model was updated in several ways, increasing in complexity. It was reasoned that the inclusion of a new predictor should require re-estimation of the model coefficients. This, as well as the model comparison results, made model 5 the best choice of models 2–5. The inclusion of ethnicity resulted in ethnicity interactions with eGFR and ACR also being included in the model. Previous studies have found ACR and eGFR are differentially associated with CKD progression across ethnicities, which indicates the interactions are clinically feasible rather than data driven [25–27].

When assessing models 5 and 6 in a competing risks setting, it was apparent that risk of death affected predicted risk of KRT. Model 6 was therefore the best choice as an updated KFRE.

Other studies have shown the KFRE overestimates 5-year risk of needing KRT in the real-world setting [14, 17]. Previously, a high-risk cohort has been used. This study is the first to illustrate the need for a competing risks model in a primary care cohort, with a risk range more representative of the general CKD population.

The clinical impact assessment compared each model and the previous NICE guidelines of referral (if *eGFR* < 30 ml/min/1.73 ${\mathrm{m}}^{2}$ and/or *ACR* ≥ 70 mg/mmol). This was to evaluate whether the same conclusions made in Major et al. would be drawn when using the new models and separating by ethnicity [9]. All models had an increased net benefit at the 5% threshold probability compared to the NICE guidelines in the white cohort. A 5% threshold is equivalent to a clinician recommending a maximum of 20 patients for referral with 1 patient developing KRT [28]. Using this threshold, the models performed better than the NICE guidelines. Comparatively, in the South Asian cohort, models 2–4 performed worse than the NICE guidelines. This could be due to a smaller South Asian population informing the models, and thus, they are more tailored to the white population, or those of South Asian ethnicity require more predictors, such as DM, to improve model performance. For this reason, the hybrid criteria combining predicted risk ≥ 5% with *ACR* ≥ 70 mg/mmol were evaluated in the subsequent clinical impact assessment.

Models 5 and 6 performed best in both ethnicities. This superior performance is partly explained as the models were developed in this dataset. Missed referrals refer to missed cases using these criteria; in clinical practice, other criteria such as family history of ESKD and the doctor’s personal judgement would also be a factor in referral. Furthermore, model 6 showed an increased clinical usefulness over model 5. This confirmed the results from the model comparison were true in clinical practice. The UK-recalibrated KFRE could be replaced with model 6 for use in clinical practice after external validation.

Though the model performed better than the previous NICE guidelines at the 5% threshold, the guidelines showed a superior performance at other threshold probabilities. Hybrid criteria incorporating the ACR criterion as well as model 6 are recommended.

This analysis again confirmed the conclusion that the KFRE can be used in the UK as a tool for predicting risk of KRT [6]. However, including an ethnicity predictor and accounting for the competing risk of death would improve both model performance and clinical impact. Whilst there have been studies that show differences in risk of KRT between ethnicities [3, 25], no studies have investigated the performance of the KFRE by ethnicity in the UK, particularly in a white and South Asian cohort. Previous research in relation to competing risk has focused on more advanced CKD, using a multistate model to predict ESKD, mortality and cardiovascular events [29]. This study is the first to show the clinical impact of a competing risks model.

The current study had a number of strengths. The dataset was large with over 400 KRT events, allowing for a precise external validation. The sample was representative of the general CKD population. The novel findings provided evidence that ethnicity must be considered when updating the KFRE and also highlighted the importance of competing risks in a primary care setting. The analysis included an external validation, a clinical impact assessment and the development and internal validation of a Cox and Fine and Gray model rather than development of a *de novo* risk prediction tool. In particular, the clinical impact assessment provided the ability to quantify each model’s usefulness in the real world.

There were some limitations to the study. Although the cohort was representative, it was geographically restricted to the East Midlands and may not represent the whole UK. The South Asian population was smaller than the white population, with less KRT events, resulting in more uncertainty with model predictions in this cohort. Though models 5 and 6, the newly developed models, were internally validated, an external validation of model 6 in a larger South Asian population is needed to ensure model performance. Minimal data was available on other ethnicities, resulting in the focus on South Asian ethnicity. Finally, the cohort was formed on the basis of all individuals having a recorded eGFR and ACR, so particularly for the latter, some bias may have occurred by excluding individuals where testing of this had not occurred.

These results provide motivation for further research into multiple areas of study. The findings from this analysis may extend to patients of other ethnicities. Further research could consider other ethnicities, as there is evidence that other ethnic minority groups have different rates of CKD progression [26, 27]. Additionally, other predictors could be considered for inclusion after adding ethnicity. This was not done when updating the model as the KFRE was developed as a simple tool for use in the general population. Similarly, to keep models 5 and 6 comparable, the same predictors were used, rather than repeating the model building process in a competing risks setting.

## Conclusions

This study has found that patients of South Asian ethnicity have a greater risk of KRT than those of white ethnicity, and the KFRE needs to consider this. Additionally, the KFRE should be updated to a competing risks model to account for risk of death. These changes could potentially have an important impact on the clinical utility of KFRE and reduce unnecessary referrals from primary care to secondary care kidney services.

### Supplementary Information

**Additional file 1: Supplementary Fig. 1.** Kaplan-Meier ESKD-free curves for each risk group, by ethnicity. **Supplementary Fig. 2.** The observed risk of KRT found using the Kaplan-Meier estimates and Aalen-Johansen estimates. **Supplementary Fig. 3.** Calibration plots for models 5 and 6 using Aalen-Johansen estimates of incidence of KRT within 5 years.**Supplementary Fig. 4.** Scatter plot of 5-year predicted risk according to models 5 and 6. **Supplementary Table 1.** TRIPOD Checklist. **Supplementary Table 2.** A comparison of the updates made to models 2-4. **Supplementary Table 3.** Model selection for model 5. **Supplementary Table 4.** Optimism-adjusted coefficients in models 5 and 6. **Supplementary Text 1.** Non-North American Kidney Failure Risk Equation for 5-year risk. **Supplementary Text 2.** Model equations for models 2-5. Model 2a (white cohort) – update 5-year baseline hazard. Model 2b (South Asian cohort) – update 5-year baseline hazard. Model 3a (white cohort) – update 5-year baseline hazard & scale of linear predictor. Model 3b (South Asian cohort) – update 5-year baseline hazard & scale of linear predictor. Model 4 – Addition of ethnicity as a predictor & update 5-year baseline hazard, scale of linear predictor. Model 5 – development of a new model. **Supplementary Text 3.** Prediction model equation for model 6.

## Data Availability

The dataset and R script used in the analysis of this study are available from Github (https://github.com/BRCLeicesterData/KFRE).

## Abbreviations

CKD: Chronic kidney disease
ESKD: End-stage kidney disease
KRT: Kidney replacement therapy
KFRE: Kidney failure risk equation
eGFR: Estimated glomerular filtration rate
ACR: Albumin-to-creatinine ratio
UK: United Kingdom
NICE: National Institute for Health and Clinical Excellence
TRIPOD: Transparent Reporting of a multivariable prediction model for Individual Prognosis Or Diagnosis
CKD-EPI: Chronic Kidney Disease Epidemiology Collaboration
CI: Confidence interval
DM: Diabetes mellitus
O/E: Observed/expected
