# Supplementary material for: Using the kidney failure risk equation to predict end-stage kidney disease in CKD patients of South Asian ethnicity: an external validation study
Source: Diagn Progn Res. 2023 Oct 5;7:22. doi: 10.1186/s41512-023-00157-x (PMC10552237; doi:10.1186/s41512-023-00157-x)
Supplement: Supplementary file 1 — Additional file 1: Supplementary Fig. 1. Kaplan-Meier ESKD-free curves for each risk group, by ethnicity. Supplementary Fig. 2. The observed risk of KRT found using the Kaplan-Meier estimates and Aalen-Johansen estimates. Supplementary Fig. 3. Calibration plots for models 5 and 6 using Aalen-Johansen estimates of incidence of KRT within 5 years.Supplementary Fig. 4. Scatter plot of 5-year predicted risk according to models 5 and 6. Supplementary Table 1. TRIPOD Checklist. Supplementary Table 2. A comparison of the updates made to models 2-4. Supplementary Table 3. Model selection for model 5. Supplementary Table 4. Optimism-adjusted coefficients in models 5 and 6. Supplementary Text 1. Non-North American Kidney Failure Risk Equation for 5-year risk. Supplementary Text 2. Model equations for models 2-5. Model 2a (white cohort) – update 5-year baseline hazard. Model 2b (South Asian cohort) – update 5-year baseline hazard. Model 3a (white cohort) – update 5-year baseline hazard & scale of linear predictor. Model 3b (South Asian cohort) – update 5-year baseline hazard & scale of linear predictor. Model 4 – Addition of ethnicity as a predictor & update 5-year baseline hazard, scale of linear predictor. Model 5 – development of a new model. Supplementary Text 3. Prediction model equation for model 6. [file 41512_2023_157_MOESM1_ESM.docx]

**Supplementary Table 1** - TRIPOD Checklist

| **Section/Topic** | **Item** |  | **Checklist Item** | **Page** |
| --- | --- | --- | --- | --- |
| **Title and abstract** | | | | |
| Title | 1 | D;V | Identify the study as developing and/or validating a multivariable prediction model, the target population, and the outcome to be predicted. | Title |
| Abstract | 2 | D;V | Provide a summary of objectives, study design, setting, participants, sample size, predictors, outcome, statistical analysis, results, and conclusions. | Abstract |
| **Introduction** | | | | |
| Background and objectives | 3a | D;V | Explain the medical context (including whether diagnostic or prognostic) and rationale for developing or validating the multivariable prediction model, including references to existing models. | Intro; paragraphs 1-3 |
|  | 3b | D;V | Specify the objectives, including whether the study describes the development or validation of the model or both. | Intro; paragraph 6 |
| **Methods** | | | | |
| Source of data | 4a | D;V | Describe the study design or source of data (e.g., randomized trial, cohort, or registry data), separately for the development and validation data sets, if applicable. | Methods; paragraph 1 |
|  | 4b | D;V | Specify the key study dates, including start of accrual; end of accrual; and, if applicable, end of follow-up. | Methods; paragraph 1 |
| Participants | 5a | D;V | Specify key elements of the study setting (e.g., primary care, secondary care, general population) including number and location of centres. | Methods; paragraph 1 |
|  | 5b | D;V | Describe eligibility criteria for participants. | Methods; paragraph 1 |
|  | 5c | D;V | Give details of treatments received, if relevant. | Not relevant |
| Outcome | 6a | D;V | Clearly define the outcome that is predicted by the prediction model, including how and when assessed. | Methods; paragraph 1 |
|  | 6b | D;V | Report any actions to blind assessment of the outcome to be predicted. | NA |
| Predictors | 7a | D;V | Clearly define all predictors used in developing or validating the multivariable prediction model, including how and when they were measured. | Methods; paragraphs 1-2 |
|  | 7b | D;V | Report any actions to blind assessment of predictors for the outcome and other predictors. | NA |
| Sample size | 8 | D;V | Explain how the study size was arrived at. | Methods; paragraphs 1,3 |
| Missing data | 9 | D;V | Describe how missing data were handled (e.g., complete-case analysis, single imputation, multiple imputation) with details of any imputation method. | Methods; paragraph 4 |
| Statistical analysis methods | 10a | D | Describe how predictors were handled in the analyses. | Methods; paragraph 4, supporting information |
|  | 10b | D | Specify type of model, all model-building procedures (including any predictor selection), and method for internal validation. | Methods; paragraphs 9, 12 |
|  | 10c | V | For validation, describe how the predictions were calculated. | Methods; paragraph 5,  supporting information |
|  | 10d | D;V | Specify all measures used to assess model performance and, if relevant, to compare multiple models. | Methods; paragraphs 6-7 |
|  | 10e | V | Describe any model updating (e.g., recalibration) arising from the validation, if done. | Methods; paragraph 8, Table 1 |
| Risk groups | 11 | D;V | Provide details on how risk groups were created, if done. | Methods; paragraph 5 |
| Development vs. validation | 12 | V | For validation, identify any differences from the development data in setting, eligibility criteria, outcome, and predictors. | Results; Table 2 caption |
| **Results** | | | | |
| Participants | 13a | D;V | Describe the flow of participants through the study, including the number of participants with and without the outcome and, if applicable, a summary of the follow-up time. A diagram may be helpful. | Results; paragraph 2, Table 2 |
|  | 13b | D;V | Describe the characteristics of the participants (basic demographics, clinical features, available predictors), including the number of participants with missing data for predictors and outcome. | Results; paragraph 1-2, Table 2 |
|  | 13c | V | For validation, show a comparison with the development data of the distribution of important variables (demographics, predictors and outcome). | Results; Table 2 caption |
| Model development | 14a | D | Specify the number of participants and outcome events in each analysis. | Results; Table 2 |
|  | 14b | D | If done, report the unadjusted association between each candidate predictor and outcome. | NA |
| Model specification | 15a | D | Present the full prediction model to allow predictions for individuals (i.e., all regression coefficients, and model intercept or baseline survival at a given time point). | Supporting information |
|  | 15b | D | Explain how to the use the prediction model. | Supporting information |
| Model performance | 16 | D;V | Report performance measures (with CIs) for the prediction model. | Results; Paragraphs 3-10, Table 3, Figures 1-2, Supporting information |
| Model-updating | 17 | V | If done, report the results from any model updating (i.e., model specification, model performance). | Results; Paragraph 7, Table 4, 6, Supporting information |
| **Discussion** | | | | |
| Limitations | 18 | D;V | Discuss any limitations of the study (such as nonrepresentative sample, few events per predictor, missing data). | Discussion; Paragraph 10 |
| Interpretation | 19a | V | For validation, discuss the results with reference to performance in the development data, and any other validation data. | Discussion; Paragraphs 5,6,9 |
|  | 19b | D;V | Give an overall interpretation of the results, considering objectives, limitations, results from similar studies, and other relevant evidence. | Discussion |
| Implications | 20 | D;V | Discuss the potential clinical use of the model and implications for future research. | Discussion; paragraphs 5-7, 11 |
| **Other information** | | | | |
| Supplementary information | 21 | D;V | Provide information about the availability of supplementary resources, such as study protocol, Web calculator, and data sets. | Supporting information |
| Funding | 22 | D;V | Give the source of funding and the role of the funders for the present study. | Funding |

**Supplementary Table 2 – A comparison of the updates made to models 2-4.**

|  | Original KFRE | Model 2a | | Model 2b | Model 3a | | Model 3b | Model 4 |
| --- | --- | --- | --- | --- | --- | --- | --- | --- |
|  |  | *White* | *South Asian* | | *White* | *South Asian* | |  |
| Baseline survival | 0.9365 | 0.9605 | 0.9389 | | 0.9614 | 0.9306 | | 0.9605 |
| Adjustment factor | 1 | 1 | 1 | | 1.0298 | 0.9188 | | 0.9994 |
| South Asian coefficient (95% CI) | -- | -- | -- | | -- | -- | | 0.4435 (0.250, 0.637) |

*Included are: baseline survival, linear predictor adjustment factor, β coefficient (corresponding to the log hazard ratio) for ethnicity with 95% confidence interval. Ethnicity was coded as a categorical variable with white as the reference category. The original KFRE and models 2a, 2b, 3a, 3b did not include a coefficient for ethnicity.*

*KFRE; Kidney Failure Risk Equation CI; confidence interval*

**Supplementary Table 3 – Model selection for model 5**

| Model | Interaction with ethnicity | Log-likelihood | Difference in log-likelihood | P-value |
| --- | --- | --- | --- | --- |
| Sex, Age, ACR, eGFR, Ethnicity |  | -3942.5 |  |  |
|  | Age | -3942.3 | 0.2 | 0.545 |
|  | Sex | -3942.5 | 0.0 | 0.903 |
|  | ACR | -3940.4 | 2.1 | **0.0397** |
|  | eGFR | -3940.4 | 2.1 | 0.0415 |
| + Ethnicity*ACR | Age | -3939.7 | 0.3 | 0.243 |
|  | Sex | -3940.4 | 0.0 | 0.961 |
|  | eGFR | -3935.9 | 4.5 | **0.0026** |
| + Ethnicity*eGFR | Age | -3935.0 | 0.9 | 0.184 |
|  | Sex | -3935.9 | 0.0 | 0.907 |

Likelihood ratio tests used for model selection. Interactions between ACR and ethnicity and eGFR and ethnicity were found to be significant. Values in bold indicate a p-value <0.05 and thus the addition of a new term.

ACR; albumin-to-creatinine ratio eGFR; estimated glomerular filtration rate

**Supplementary Table 4 – Optimism-adjusted coefficients in models 5 and 6**

| Covariate | Model 5 coefficients | Model 6 coefficients | Original KFRE coefficient |
| --- | --- | --- | --- |
| Baseline survival | 0.9975 | 0.9650 | 0.9570 |
| Age | -0.5584 | -0.5562 | -0.2201 |
| eGFR | -0.5006 | -0.4131 | -0.5567 |
| ACR | 0.5626 | 0.4382 | 0.4510 |
| Male | 0.5339 | 0.4429 | 0.2467 |
| South Asian | 0.1838 | 0.2655 | -- |
| ACR*South Asian | 0.2370 | 0.1894 | -- |
| eGFR*South Asian | 0.1233 | 0.0943 | -- |

The coefficients of the original KFRE are also reported for comparison.

ACR; albumin-to-creatinine ratio eGFR; estimated glomerular filtration rate KFRE; Kidney Failure Risk Equation

**Supplementary Figure 1 – Kaplan-Meier ESKD-free curves for each risk group, by ethnicity**


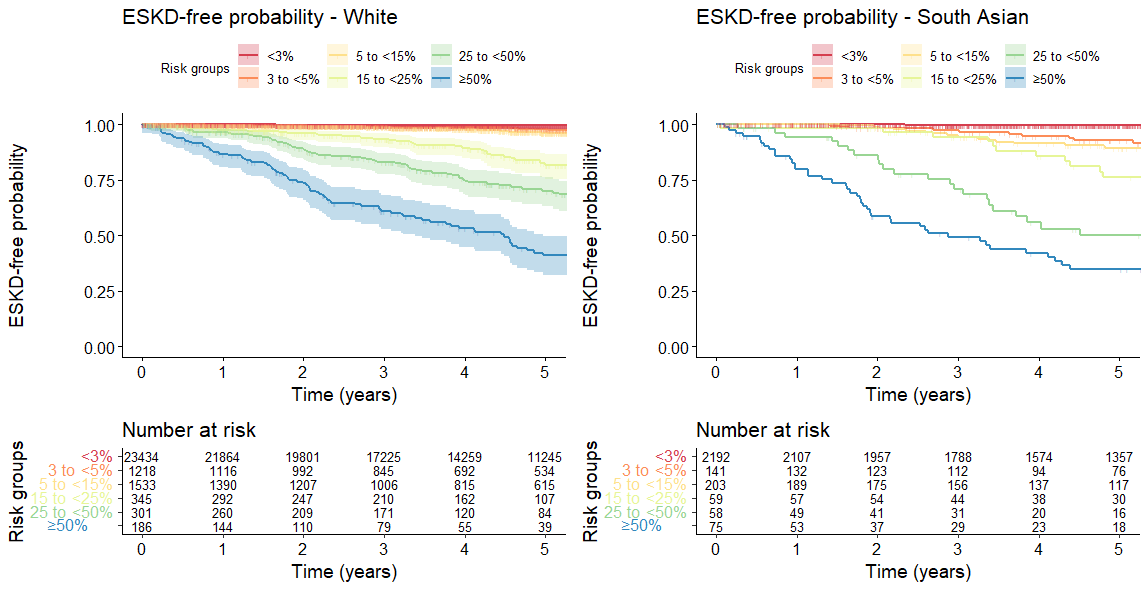


The risk table shows number at risk of ESKD, therefore needing KRT, in each group at yearly intervals.

ESKD; end-stage kidney disease

**Supplementary Figure 2 – The observed risk of KRT found using the Kaplan-Meier estimates and Aalen-Johansen estimates**

Kaplan-Meier estimates are dashed lines and Aalen-Johansen estimates are solid lines. The lines were separated by risk group to illustrate separation of risk more clearly.

KRT; kidney replacement therapy


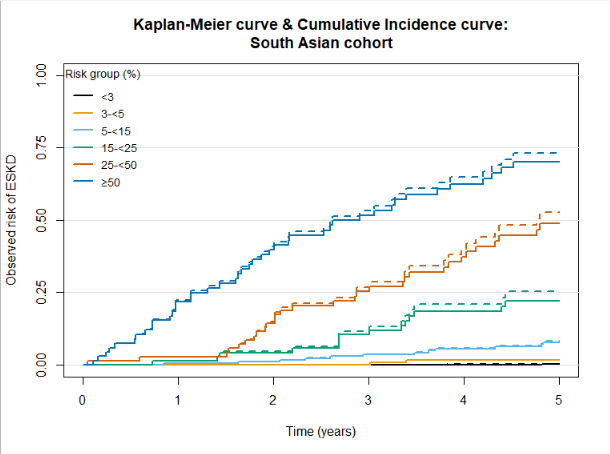

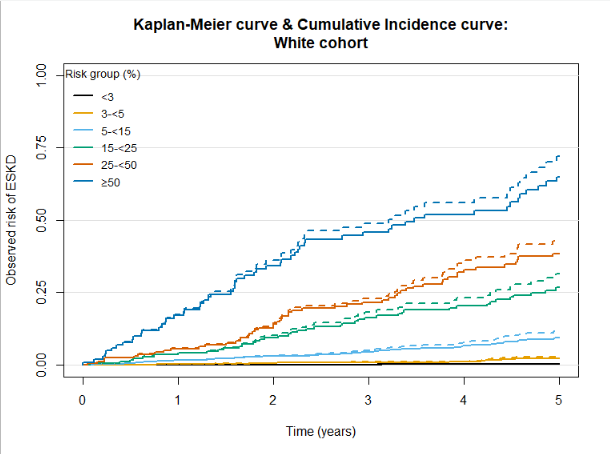


**Supplementary Figure 3 – Calibration plots for models 5 and 6 using Aalen-Johansen estimates of incidence of KRT within 5 years**


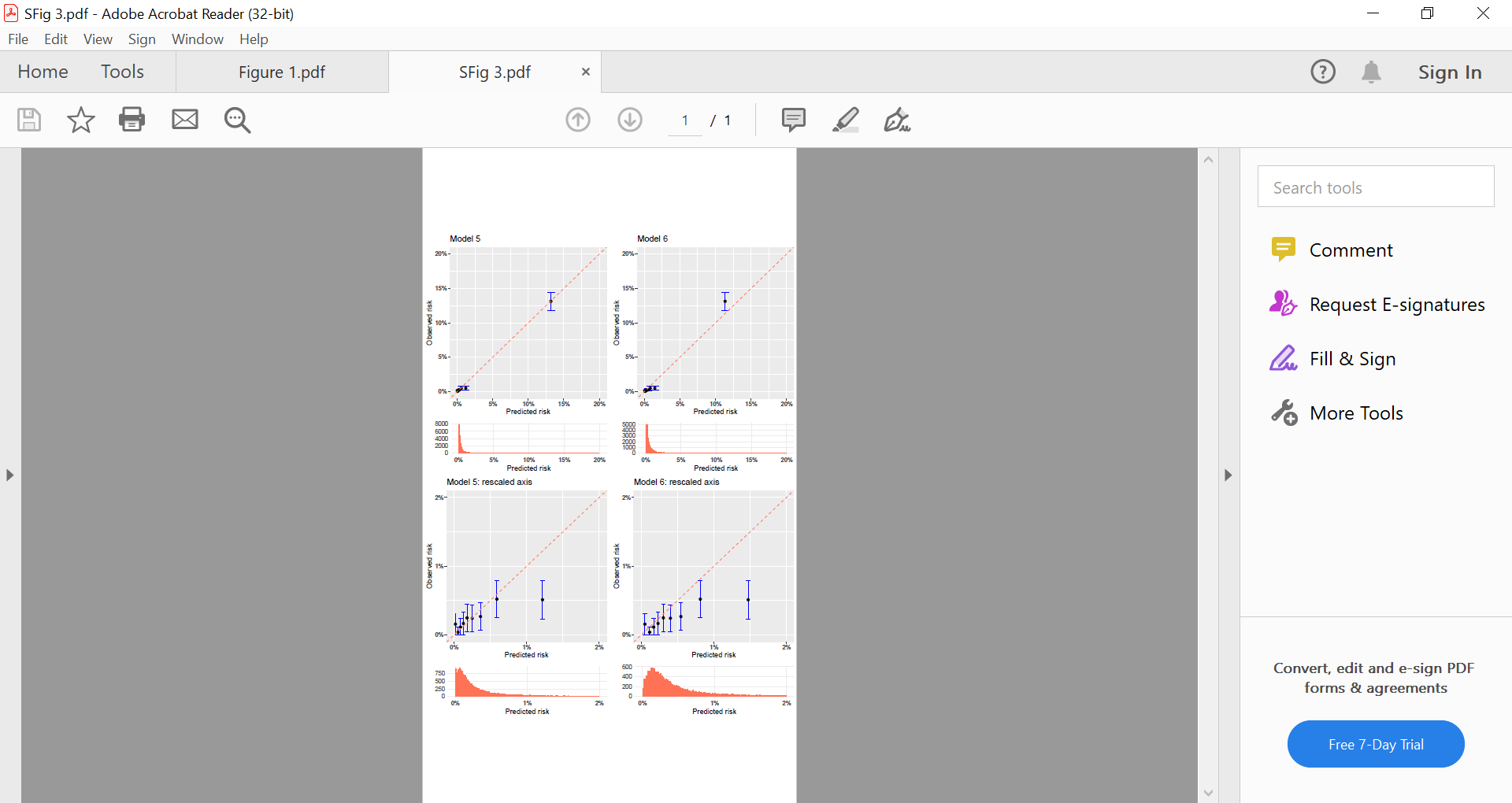


Histograms beneath the plots show the distribution of the linear predictor. The range of risk was very low, so the first row of plots were truncated at 20%, and the second row at 2%, to allow for closer inspection.

**Supplementary Figure 4 – Scatter plot of 5-year predicted risk according to models 5 and 6**


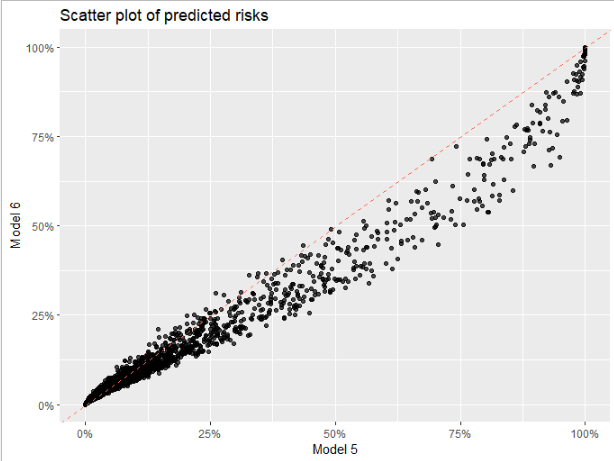


The dashed line indicates perfect agreement between the models.

**Supplementary Text 1 - Non-North American Kidney Failure Risk Equation for 5-year risk**

The risk of KRT using the KFRE 4-variable equation calibrated to a non-North American population is calculated as:

1 - 0.9365^exp(βsum)^

Where βsum = -0.2201 × (age/10 – 7.036)

+ 0.2467 × (male – 0.5642)

– 0.5567 × (eGFR/5 – 7.222)

+ 0.4510 × (logACR – 5.137)

KRT; kidney replacement therapy eGFR; estimated glomerular filtration rate ACR; albumin-to-creatinine ratio

**Supplementary Text 2 – Model equations for models 2-5**

Further information on model updates is seen by providing model equations, which highlights the difference in each model from the non-North American KFRE.

**Model 2a (white cohort) – update 5-year baseline hazard**

1 - 0.9605^exp(βsum)^

Where βsum = -0.2201 × (age/10 – 7.036)

+ 0.2467 × (male – 0.5642)

– 0.5567 × (eGFR/5 – 7.222)

+ 0.4510 × (logACR – 5.137)

**Model 2b (South Asian cohort) – update 5-year baseline hazard**

1 - 0.9389^exp(βsum)^

Where βsum = -0.2201 × (age/10 – 7.036)

+ 0.2467 × (male – 0.5642)

– 0.5567 × (eGFR/5 – 7.222)

+ 0.4510 × (logACR – 5.137)

**Model 3a (white cohort) – update 5-year baseline hazard & scale of linear predictor**

1 - 0.9306^exp(βsum)^

Where βsum = 1.030 × [-0.2201 × (age/10 – 7.036)

+ 0.2467 × (male – 0.5642)

– 0.5567 × (eGFR/5 – 7.222)

+ 0.4510 × (logACR – 5.137)]

**Model 3b (South Asian cohort) – update 5-year baseline hazard & scale of linear predictor**

1 - 0.9614^exp(βsum)^

Where βsum = 0.9188 × [-0.2201 × (age/10 – 7.036)

+ 0.2467 × (male – 0.5642)

– 0.5567 × (eGFR/5 – 7.222)

+ 0.4510 × (logACR – 5.137)]

**Model 4 – Addition of ethnicity as a predictor & update 5-year baseline hazard, scale of linear predictor**

1 - 0.9605^exp(βsum)^

Where βsum = 0.9994 × [-0.2201 × (age/10 – 7.036)

+ 0.2467 × (male – 0.5642)

– 0.5567 × (eGFR/5 – 7.222)

+ 0.4510 × (logACR – 5.137)

+ 0.4435 × South Asian]

**Model 5 – development of a new model**

1 - 0.9975^exp(βsum)^

Where βsum = – 0.5584 × (age/10 – 7.036)

+ 0.5339 × (male – 0.5642)

– 0.5006 × (eGFR/5 – 7.222)

+ 0.5626 × (logACR – 5.137)

+ 0.1838 × South Asian

+ 0.1233 × South Asian × (eGFR/5 – 7.222)

+ 0.2370 × South Asian × (logACR – 5.137)

KRT; kidney replacement therapy eGFR; estimated glomerular filtration rate ACR; albumin-to-creatinine ratio

**Supplementary Text 3 - Prediction model equation for model 6**

The risk of KRT within 5 years using model 6 (a Fine and Gray model) can be calculated as follows:

1 - 0.9650^exp(βsum)^

Where βsum = -0.5662 × (age/10 – 7.036)

+ 0.4429 × (male – 0.5642)

– 0.4131 × (eGFR/5 – 7.222)

+ 0.4382 × (logACR – 5.137)

+ 0.2655 × South Asian

+ 0.0943 × South Asian × (eGFR/5 – 7.222)

+ 0.1894 × South Asian × (logACR – 5.137)

KRT; kidney replacement therapy eGFR; estimated glomerular filtration rate ACR; albumin-to-creatinine ratio
